# Innate Immunity Stimulation during the COVID-19 Pandemic: Challenge by Parvulan

**DOI:** 10.1155/2022/4593598

**Published:** 2022-04-29

**Authors:** Beniamino Palmieri, Antonio Manenti, Francesca Galotti, Maria Vadalà

**Affiliations:** ^1^Medico Cura Te Stesso Onlus, Modena, Italy; ^2^Second Opinion Medical Network, Modena, Italy; ^3^Department of Surgery, University of Modena, Italy; ^4^Department of Anaesthesiology and Reanimation, University of Torino, Italy

## Abstract

**Aim:**

We report an open spontaneous anecdotical retrospective survey of *Corynebacterium parvum* administration to 4000 fragile immune-depressed and multimorbid patients treated with a killed *C. parvum* strain to enhance innate immunity, integrating the adaptative immune response for long-standing antinfectious resistance.

**Methods:**

A total of 4000 patients (1900 men and 2100 women) with mild, moderate, or chronic disease, appealing to our Second Opinion Medical Consultation Network, signed an informed consent form and were injected subcutaneously with *C. parvum*. The treatment was followed up to 6 months, completing the short form of the medical outcome health survey questionnaire (SF-36) directly by the patients or their parents and monitoring their health status regularly via telemedicine (Skype, WhatsApp, mail, etc.) or outpatients visits.

**Results:**

The main efficacy endpoints, as assessed by the SF-36 questionnaire are: significant improvements in the mental and physical role functioning score (*p* < 0.02), better general health; social role performance (*p* < 0.02), vitality (*p* < 0.03), and a significant pain reduction (*p* < 0.03). A quick (48-72 hours) symptoms improvement and/or complete regression of the herpetic eruptions was observed in 1000 affected patients with disappearance or relieve of herpetic neuralgia (reduced in 80% of cases); also full recovery or frequency reduction (30%) of recurrent cystitis and prostatitis in 120 affected patients. Last but not least, a life quality improvement in 100 oncologic patients of overall 200 cases. A significant increase in the lymphocyte count (*p* < 0.01), mainly helper and killer lymphocytes, was noted 6 months after Parvulan injection vs. the baseline. The asymptomatic SARS-CoV-2 patients who were incidentally enrolled in our survey were tested at the sixth month for antibodies against SARS-CoV-2, and 14 patients had high levels of SARS-CoV-2 antibodies. The incubating COVID infections of the Parvulan-injected patients even if frail and multimorbid recovered in short term (48-96 hours) with a benign clinical course, without need of drugs administration except for the variants, such as Delta and Omicron, whose infections lasted on average one week and required some antipyretics and low-dose steroids for a few days.

**Conclusions:**

Our results confirm that *C. parvum* is quite safe and effective in supporting immune-compromised patients when epidemic or pandemic events increase the life risk and any kind of infection and complication rate. Further double-blind placebo evidence-based studies are urgently required, and our numerically substantial not sponsored spontaneous observation is exclusively intended to promote further evidence-based double-blind institutional studies.

## 1. Introduction

Innate immunity and adaptive immunity are the two milestones of human defence against pathogenic agents facing either single patient infection or epidemic/pandemic widespread infections [1–4].

Innate immunity is the quick answer of five phagocytizing cytotypes ([namely, monocytes, macrophages, dendritic cells, antigen-presenting cells (APCs), metamyelocytes, and killer lymphocytes) [5–7]. They fight any noxious invading agents at entrance sites (viruses, bacteria, fungi, and protozoa) and scavenge the bloodstream. In fact, the innate immune system uses pattern recognition receptors, including Toll-like receptors (TLRs), CD209+, mannose receptors, and complement receptors, to help recognize and kill invading pathogens [8]. Meanwhile, adaptive immunity, through dendritic and T and B cell memory activation, is alerted and starts antibody production more slowly [9].

The clearance of viruses, bacteria, and fungi with activation of interferon family genes is usually completed in 48-72 hours; the crosstalk between T and B immunity is contemporarily started to synthetize targeted antibodies in 2-4 weeks, with a long memory against possible future relapses [10, 11]. The synergy of the two arms of immunity is also effectively integrated in terms of balanced antibody production and prevention of the wrong autoimmune response [12, 13].

*Corynebacterium parvum* (recently renamed *Cutibacterium acnes*) is a millenarian commensal of the human skin that checks and balances its microflora environment, whose immune modulating properties were discovered in the 1960s, burying the killed bacterium into the subcutis [14–16].

The very first clinical use of *C. parvum* as a drug (Coryparv, by the International Wellcome Burroughs Company) was in the oncological setting because of its direct anticancer properties (cancer tissue infiltration of lymphomonocytes and macrophages) with prolonged survival and better quality of life [17–19].

During our oncological studies, we realized that some patients contemporarily affected by sudden viral infections remitted the infectious symptoms in 2-4 days, and these serendipitous observations were by us followed up and extended over the years to cure different common virus infections either in otherwise healthy or in multimorbid sick people [8, 9, 17, 20–22].

Meanwhile, many preclinical microbiological and virological studies supported this anti-infectious *C. Parvum* properties, but the registration of Coryparv expired in Europe, and nobody else, except us, growing the original strain in the university lab, pursued anecdotally to use it safely in viral, bacterial, and fungal resistant infections.

During the impending pandemic in 2020, before the distribution of specific vaccines, aside from the government recommendations to stimulate the trained immunity by repeating previous vaccinations (mumps varicella, TB, and influential), we remembered the previous *C. parvum* experience and started primarily self-administration on us among a group of practicing physicians with Parvulan (Brazilian brand Extratos Alergenicos), the unique worldwide ongoing original registration [9, 23, 24].

Subsequently, proved the benefits on the innate immune system by our lab tests, we could achieved the authorization to extend the practice also to selected high-risk people and frail multimorbid immunocompromised patients with infectious diathesis mainly in oncology and geriatrics.

## 2. Materials and Methods

Our study is a spontaneous anecdotal, observational, retrospective investigation involving every Italian region, namely, Veneto, Piemonte, Lombardy, and Emilia Romagna.

**Figure 1:**
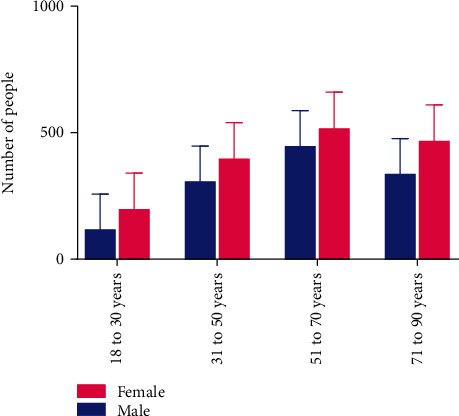
Distribution of population by age and sex. The bar graph shows the numbers of males and females in the 4-year age groups.

A total of 4000 patients (1900 men and 2100 women) with diagnosed mild, moderate, or severe diseases, aged 18-90 years, who were referred to our Second Opinion Medical Consultation Network∗ between November 2020 and May 2021, were admitted to Parvulan injection **(**Table 1, Figure 1).

The Second Opinion Medical Network is a consultation referral web and outpatient office system enclosing a wide panel of specialists, to whom any patient with whatever illness or syndrome inadequately faced by the diagnosis and therapy can apply for an individual clinical audit [25–28].

The clinical data of the recruited patients are described in Table 2.

### 2.1. Inclusion and Exclusion Criteria

The inclusion criteria were as follows: patients complaining of chronic recurrent mild/moderate/severe bacterial and viral or fungal infections in the previous 6 months and seeking effective and adequate therapy beyond antibiotics. They were recruited among the following cohorts: cancer, primary, secondary, and iatrogenic immunosuppression and multiple comorbidities, including old and very old people.

Being the start-up of our prescription coincident with the pandemic phase of SARS-CoV-2 from 2020 to the first semester of 2021, we could not rule out the inclusion of symptomatic or asymptomatic COVID-19-infected patients, generally in sustainable clinical conditions and exclusively at very early symptom arousal (max 2-3 days of incubation), with a saturation index higher than 85% and good hemodynamic and hematological parameters. The exclusion criteria included hospitalized patients, subjects with multiorgan failure, autoimmune diseases, pregnant women, terminal cancer patients, cachexia, and patients with severely compromised cardiorespiratory and renal insufficiency.

Two office physicians at the Second Opinion Medical Network facility monitored the group daily by phone, mail, Skype, WhatsApp, or outpatient visits. The clinical follow-up and side effects to the drug treatment were overviewed with monthly auditing in terms of safety, effectiveness, and compliance of subjects.

### 2.2. Clinical Protocol of Subcutaneous Injection of Parvulan

Each patient signed an informed consent of spontaneous submission to the procedure that was shipped to the Italian Medicines Agency (AIFA), accomplishing the rule of Institutional control of a drug registered in a foreign nation (by ANVISA Brazil) outside of the European Medicines Agency (EMA), to be injected in full accordance with the registered drug leaflet.

The post injection treatment was followed up to 6 months, filling the pre/post short form of the medical outcome health survey questionnaire (SF-36), directly by the patients or their nurses/relatives. It measures health-related quality of life (QoL) in eight settings: vitality, general health perceptions, physical functioning, physical role functioning, emotional role functioning, social role functioning, bodily pain, and mental health. Each scale is scored using norm-based methods, with percentage scores ranging from 0% (lowest or worst response) to 100% (highest or best possible response) [29–31].

### 2.3. Parvulan Preparation, Dosage, and Administration

A total of 2.5 cc of the Parvulan ampoule content was mixed with 0.25 cc of 1% xylocaine (to perform a painless injection) and 0.25 cc of low molecular weight (4000 Dalton) recombinant hyaluronic acid **(**commercially available in the dermoaesthetics area) to promote a hydrophilic environment around the injected core to enhance the recruiting and imprinting of chemotactically challenged immune cells.

**Figure 2:**
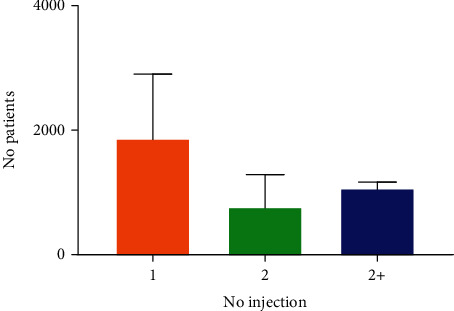
Graphic of no Parvulan injection per patient. Frequencies of Parvulan injection (once only, or twice or more) in enrolled patients (*n* = 4000), based on medical judgement and disease severity.

Parvulan was injected once only, or rarely twice, or more based on medical judgement and disease severity; the injected surface was the forearm, lateral shoulder area, and, more rarely, the upper external gluteus or lower abdominal quadrants **(**Figure 2**)**.

The patients were informed that the individual reaction to injection might cause in the first 24-48 hours some erythema or temporary itchy nodules spontaneously resolving or counteracted by antihistaminic or anti-inflammatory creams; the tuberculin like reaction is quite slight and never ulcerates. Very rarely (due to the low bacterial concentration and the tailored formulation), a slight increase of body temperature has been observed at 12 hours posttreatment, remitting spontaneously remitting, usually without antipyretics.

### 2.4. Statistical Analysis

Statistical analysis was performed using GraphPad Prism 7 (GraphPad Software Inc., San Diego, CA, USA). All data are presented as the mean ± standard deviation (SD) and were analyzed using two-way ANOVA. *p* < 0.05 was considered significant.

## 3. Results

**Figure 3:**
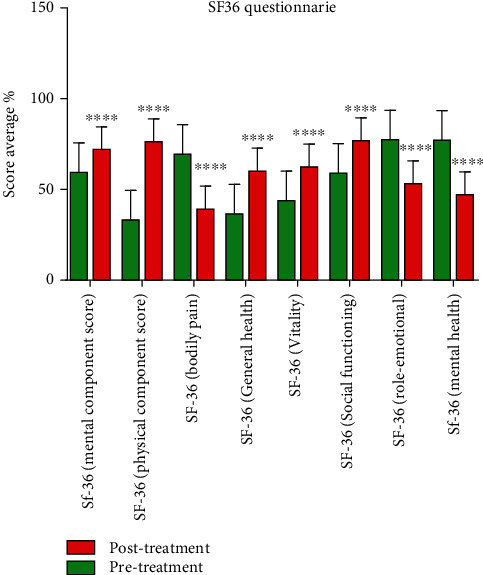
Graphical representation of the SF36 questionnaire results. Bar graphs showing the mean % SF-36 questionnaire results for the pretreatment (green graphs) and posttreatment (orange graphs) groups. The post-Parvulan injection scores were already statistically better than those collected pretreatment. Data are presented as the mean ± standard deviation (SD). There were significant differences between pre- and posttreatment. ^∗∗∗∗^*p* < 0.0001, pre- vs. posttreatment.

All the patients enrolled were treated with Parvulan. The main efficacy endpoints, as assessed by the SF-36 questionnaire administered at baseline and 3 months after treatment, are significant improvements in the mental and physical role functioning score (*p* < 0.02), in general health, in social role functioning (*p* < 0.02), in vitality (*p* < 0.03), and in a significant pain reduction (*p* < 0.03). Changes in psycho-social behavior (*p* = 0.02) or emotional state, with a reduction in typical symptoms observed during this pandemic era, including hypochondria, anxiety, panic, depression, and mood alteration, were found **(**Figure 3**).**

Using structured telephone or telematic interviews (mail, Skype, and WhatsApp) in our follow-up, we observed significant positive clinical outcomes in all the cohorts of patients, which are summarized in Table 3.

Quick improvement (72-96 hours) and complete regression of the herpetic eruptions were observed in 1000 (25%) enrolled patients; zoster neuralgia (80% of patients with herpes zoster) was improved as well recurrent cystitis and prostatitis (30% of patients) recovered in 2-3 days after injection and a life quality improved in 100 oncologic patients.

**Figure 4:**
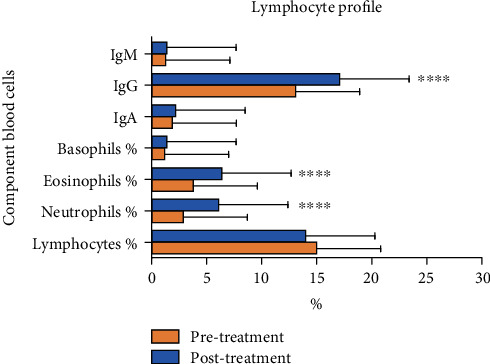
Graphical representation of lymphocyte component blood cells. The % number of IgM, IgG, IgA, basophils, eosinophils, neutrophils, and lymphocytes. There were significant differences in terms of IgG, eosinophils, and neutrophils between pre- and posttreatment. ^∗∗∗∗^*p* < 0.0001, pre- vs. posttreatment.

A significant increase in the lymphocyte count (*p* < 0.01), mainly helper and killer lymphocytes, was detected after 6 months by Parvulan injection vs. the baseline. Relevant changes in neutrophil and eosinophil counts were identified (*p* > 0.05) (Figure 4). In addition, Parvulan treatment had no influence on all other values, such as hemoglobin (Hb), alanine aminotransferase (ALT), and aspartate aminotransferase (AST) levels.

**Figure 5:**
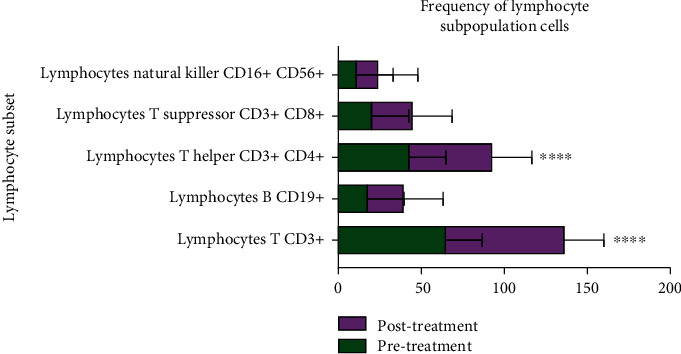
Graphical representation of lymphocyte subsets before and after treatment. Data are expressed as the mean ± standard deviation (SD). Differences in CD3+ lymphocytes and CD3+ and CD4+ T helper cells were considered statistically significant relative to the pretreatment group. ^∗∗∗∗^*p* < 0.0001, pre- vs. posttreatment.

Levels of lymphocyte subpopulations were compared before and after treatment, including CD3+ lymphocytes, T helper cells (CD3+, CD4+), suppressor T cells (CD3+, CD8+), and natural killer T cells (CD16+, CD56+) (Figure 5).

**Figure 6:**
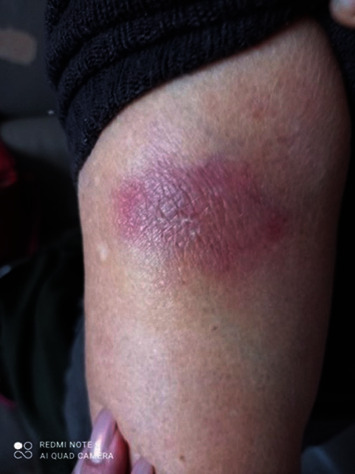
Skin rash 2 days after Parvulan injection. Improvement of the lesion after 10 days with the use of diclofenac gel.

**Figure 7:**
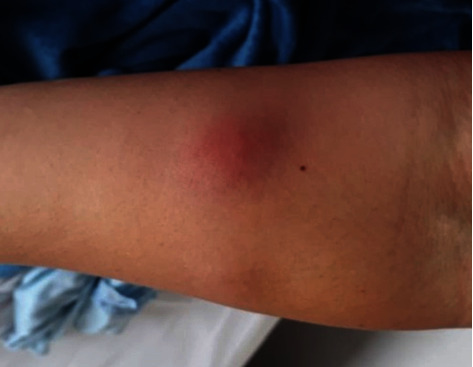
Skin rash 1 day after treatment with spontaneous regression.

**Figure 8:**
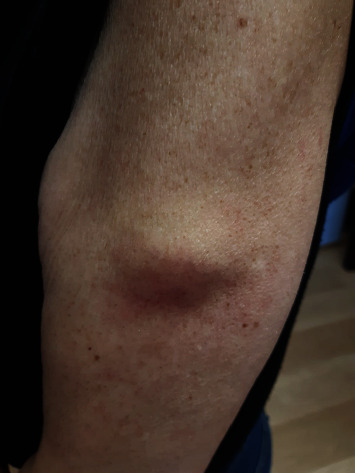
Skin lump 72 h after Parvulan injection, which regressed spontaneously after 3 weeks.

**Figure 9:**
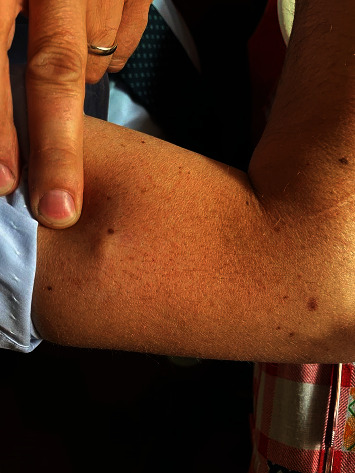
Skin lump 48 hours after treatment with spontaneous regression at 1 month.

The cutaneous reactions (rash, swelling) developed 24 to 72 hours after injection and lasted two to seven days, the skin rash resolved in 24-48 hours, and the skin soreness and lump lasted 7-10 days (1-2 months in a few cases) (Table 4) (Figures 6–9).

The skin reactions either regressed spontaneously or were successfully treated with arnica, diclofenac gel or plaster, and topic gentamycin.

The asymptomatic SARS-CoV-2 patients (3%), incidentally enrolled in our survey, were tested at the sixth month for antibodies against SARS-CoV-2, and 14 (0.35%) patients had high levels of SARS-CoV-2 antibodies.

The protection of healthy Parvulan-injected individuals was very high. SARS-CoV-2 infections in Parvulan-injected patients were short-term and had a benign clinical course (low fever, low mild oropharyngotracheobronchial symptoms, anosmia, dyspepsia, and bowel irritation) and usually did not require further drug administration except for the variants, which lasted on average a week and required some antipyretics and low-dose steroids for a few days.

The hospital admission rate was very low as well, with no deaths and no intubation required.

Regarding the anti-COVID-19 IgG IgM titers, the wide range depends on the viral load and virulence met by the Parvulan-injected patients.

## 4. Discussion and Conclusion

Our study supports the concept that the killed *C. parvum* injected subcutaneously at the proper dosages effectively activates innate immunity and offers several benefits, especially during a pandemic emergency; in fact, the brittle and multimorbid people, especially at old age, are exposed to several infectious risks contemporarily or metachronically, and each relapse or new infection increasingly weakens the healing power and vitality of the patient. Herpes simplex and zoster and aphthous mouth ulcers, for example, are sortie viruses that worsen very often the life quality of immunocompromised patients overlapping with previous infectious diseases or cancer or metabolic impairment; recurrent urinary tract infections or mycotic vaginal discharges, or pharyngotonsillitis or tracheobronchial inflammation, added to the current air pollution, relapse very frequently in sick febrile patients.

For this reason, *C. parvum* administration might putatively be considered either in prevention or in therapy aside from the current standard treatments of evidence-based medicine.

This bacterium has unique aspecific antiviral properties, quickly counteracting the invasion of many viral strains in experimental animals and human and veterinary pathology, but it has also successfully been challenged against bacteria, viruses, and protozoa by direct phagocytosis and activation of the interferon family.

Furthermore, anecdotally and incidentally, we supposed to have now retrospectively reached some proof of the concept that the una tantum treatment with *C. parvum* is effective to prevent SARS-CoV-2 infection and to neutralize the first stages (2-3 days) of the infectious symptoms of the original virus and its variants, but further controlled studies are needed to better define this issue.

Resuming the history of our *C. parvum* endorsement in clinical therapy, we were induced to use it at the beginning of the pandemic when the COVID-19 vaccines were not yet available, and the National Health Committee recommended enhancing trained immunity repeating some surrogate vaccination against, i.e., tuberculosis, influential, and mumps.

Parvulan was at that time the unique Brazilian brand registration suitable to enhance the immunity, and its use on the very first group of doctors and nurses was intended, not as a generic trained immunity challenge but much more effectively as an innate immunity enhancement against any kind of infectious agents, enclosing also SARS-CoV-2 and its variants.

The life defense of the first line emergency caregivers has been our primary practical concern to raise an effective barrier against a high spectrum of whatsoever noxious agents, reducing the overall morbidity and mortality the goal, retrospectively…. seems really to be reached…

Our study confirms that, aside from the COVID-19 vaccines, *C. parvum* treatment should not be withdrawn or dropped out.

In fact, the perfect integration between innate and adaptive immunity, accurately outlined by hundreds of scientific reports, strongly recommends to adequately blend the activation of both the arms to reach more complete, protective, and safe flock immunity.

The pre-post-Parvulan hematological biochemical evaluation performed on our patients showed a general trend to increase the blood leukocytes, especially lymphocytes, neutrophils, and monocytes and (by immune phenotyping) T helper cells and natural killer cells.

Regarding the soluble immunity titres in our cohort, the SARS-CoV-2 IgG and IgM antibody concentrations tested 2-3 months after parvulan were quite different among individuals, ranging from 0 to very high levels (1720).

We thus suppose that the very first commitment of innate immunity by parvulan is to display a palisade of immune phagocytizing cells across the infectious agents' thresholds and secondarily to alert the circulating immune cells to prevent the infection spread and sepsis; we have no routine tests available for the former local submucosa population, but we have some for the latter; therefore, the pre-post-Parvulan clinical history evaluation aside from the hematological findings is mandatory for the final judgment of effective therapy

Regarding the observed wide range of IgG SARS-CoV-2 antibodies, we reasonably suppose that they are not produced if the submucosal immune phagocytizing cells definitely prevent any virus invasion entrance, but if some virions enter in closer contact with antigen-presenting cells (APCs) locally or in the bloodstream, adaptive immunity is further triggered and modulated by the innate immune system.

A great number of our patients were affected by severe zoster infections and very quickly responded to *C. Parvum* injection; even very old neuropathic complications improved or disappeared after the treatment; in some cases, after a single Parvulan injection, the herpes relapsed more rarely on less virulent, and the injection was successfully repeated twice or three times, with lower dosages in the follow-up.

**Figure 10:**
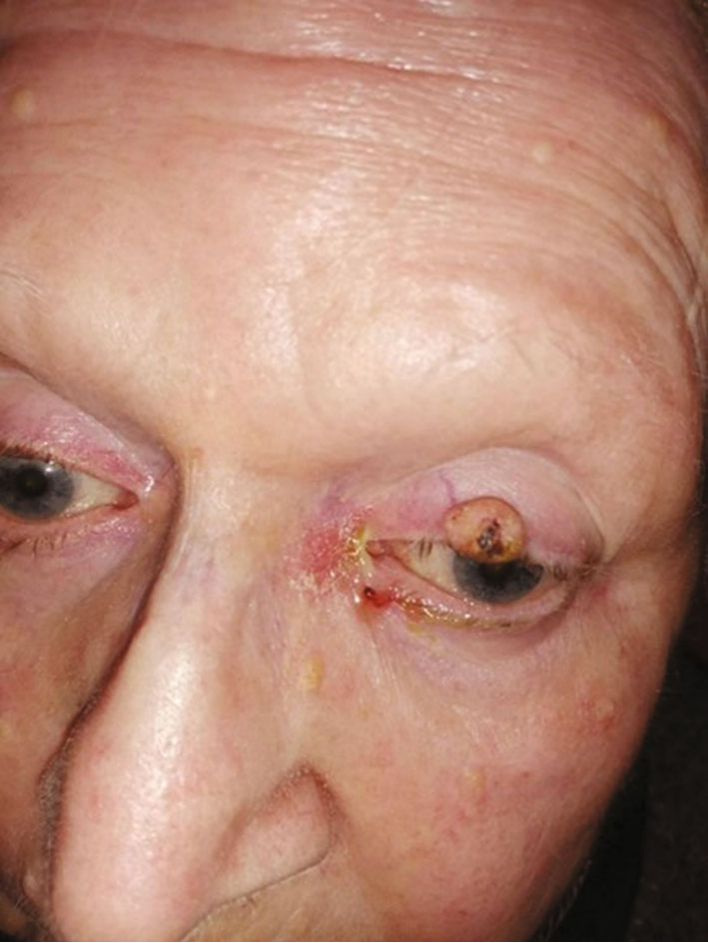
Patient (AF, 80 years) with dacryocystitis, basalioma, and blepharitis before Parvulan injection.

**Figure 11:**
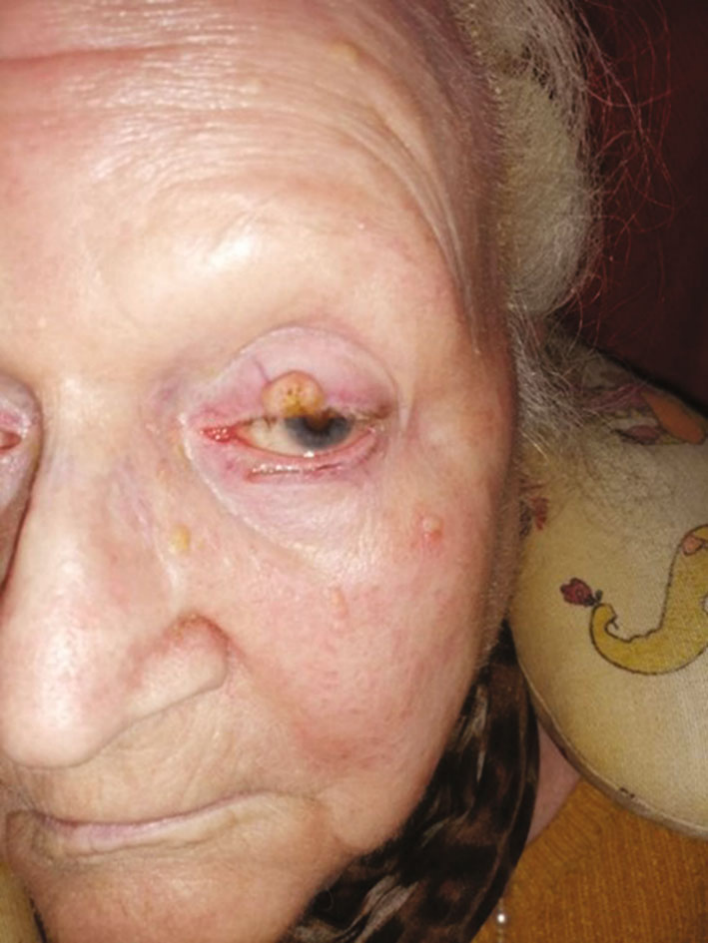
Patient (AF, 80 years) with dacryocystitis, basalioma, and blepharitis after Parvulan injection.

Other bacterial and fungal infections of skin and mucosa were successfully defeated as well (Table 3), and some inflammatory skin diseases amazingly improved (Figures 10 and 11). Incidentally, many treated patients, especially those with chronic fatigue, reported renewed brisky mental and physical energy after one week or lasting more than one month.

This note is beyond the scope of our retrospective investigation, but some literature reports claim the effects of *C. parvum*, especially on bone marrow and liver stem cells [32–35].

As to the drug safety, the 50-year-old oncological history of *C. parvum* leaves no doubt about the great compatibility of this skin millenarian commensal bacterium within human health and life; exceedingly high repeated dosages in cancer patients, adults and children (even intravenous) never caused death, anaphylactic shock, multiorgan failures, or lung cytokine storms: only fever and chills are easily controlled by antipyretics [8, 17, 21, 22]. Our experience definitelly confirms the safety of our schedule with parvulan.

In fact, our standardized dosage- and una tantum administration, confirm with exhaustive evidence the total absence of toxicity and complications, even in weakly frail patients; on the contrary, we observed some mental and physical performance improvement; the skin injection produced sometimes itchy nodules (cytologically confirmed to be lumps of lymphomonocytes chemotactically involved), quite similar to the tuberculin skin reactions that disappeared gradually and almost spontaneously.

In conclusion, our open anecdotical, spontaneous retrospective evaluation does not pretend to claim any property of *C. parvum* other than the pure declarations of the Parvulan registration leaflet;the absolute safety of this drug is confirmed by our study without any doubt or suspicion.

The activity against SARS-CoV-2 and its variant were purely incidental findings during our practice, but they have to be taken into account, as an effective opportunity, to activate the innate immunity and modulate adaptive immunity at the same time. The innate immunity in fact is the very first milestone by the survival law of every superior living species 3 million years before the development of the adoptive immunity (antibodies); the prominent role of the former, therefore, fully synergizes with the functions of the latter, also regulating some unbalanced responses of this latter.

With Parvulan, we intended to relieve the impeding life risk of frail multimorbid individuals and the goal has been reached.

We really are not aware of what judgement on our retrospective observation of 4000 treated cases, that will be sentenced by the scientific community; we are not keen to apologize for the many obvious biases that exclude ad interim our data from the evidence-based arena; we just want to stress that during a planetary emergency, some reasonably safe and useful therapeutic weapon can profitably be exhumed and individually used, without conflicting with the health government guidelines and fully accomplishing with the military vaccination strategies or other new incoming tailored treatments

Last, but not least, even if the fact that doctors preliminarily challenged on themselves the feasibility and benefits of Parvulan injection does not bring to our protocols any further strength in terms of clinical methodology and statistical survey; notwithstanding, the prior involvement of health caregivers in the study would be an ethical concern worth of attention.

Conclusively, we wish that our retrospective survey will focus the attention of clinicians and companies towards innate immunity potentiation and integration together with the adaptive one.

The historical anti-infectious and antiviral properties of *C. parvum* suggest its safe and effective use to fortify fragile patients when epidemic or pandemic events increase the life risk and complication rate.

Further double-blind placebo evidence-based studies are urgently required, and our numerically substantial not sponsored observation is aimed exclusively at this goal.

## Figures and Tables

**Table 1:** Patient clinical characteristics (sex, age, and weight). No.: number of patients; SD: standard deviation; kg: kilograms.

No. of patients	4000
Male	1900
Female	2100
Mean age (SD) (years)	38.6
Mean weight (kg)	77.8
Drug dosage, mode of treatment	3-5 mL—subcutaneous injection individually, tailored by weight and pathology

**Table 2:** Clinical-pathologic background of the treated cohort. Considering the nonnormal distribution, nonparametric statistics were used to compare the disease population. All descriptive data were reported in percentages (%).

Medical condition	*N* patients	%
Viral infection (herpes zoster, herpes simplex)	1000	25%
Virus-induced immunosuppression (multiple aetiology immune-depression)	2210	55.2%
Autoimmune disease (thyroiditis, arthritis, hypothyroidism, fibromyalgia, chronic asthenia, lupus erythematosus-SLE)	300	7.5%
Cardiovascular (hypertension, vascular brain insufficiency)	10	0.25%
Oncology (breast cancer, prostate cancer)	200	5%
Urinary tract inflammation (recurrent cystitis and prostatitis, vaginal mycosis, candida and trichomonas vaginitis, dyspareunia)	120	3%
Old age/multimorbidity/diabetes	40	1%
Healthy people (doctors, nurses, teachers)	120	3%

**Table 3:** Clinical outcome of the treatment in symptomatic patients. Effects of Parvulan injection on the no. of patients (%).

Symptom modulation	No. (%)^∗^
Quick (48-72 h) cutaneous herpetic eruption regression (thorax, head, neck, and genitals)	100%
Improvement of postherpetic zoster (neuralgia)	
Regression of common cutaneous herpes simplex (genital herpes and herpes labialis, stomatitis, and mouth sore)	80%
Delayed relapse of resolution of urinary tract inflammation (recurrent cystitis and prostatitis, vaginal mycosis, candida and trichomonas vaginitis, dyspareunia)	30%
Life quality improvement of oncologic patients	2.5%
Reduction of common symptoms in autoimmune diseases such as fibromyalgia, arthritis, psoriasis, and atopic dermatitis	10%

**Table 4:** Patients who experienced adverse events after Parvulan injection. Number (percentage) of patients with adverse events after treatment.

	*N* (%)
Skin lump and soreness at the injection site (uncomplicated, plain resolution)	1920 patients (48%)
Cutaneous swelling	320 patients (8%)
Short-term pain at the injection site	1480 patients (37%)
Injected upper arm soreness (short-term spontaneous resolution)	320 patients (8%)
Skin rash around the injected area	960 patients (24%)
Fever, low grade in the first 8 hours, no antipyretic requirement	20 patients (0.5%)
Short term fatigue (influential symptoms)	8 patients (0.2%)

## Data Availability

The authors declare that data supporting the findings of this study are available within the article.

## Additional Points

*Data Transparency*. The authors affirm that this manuscript is an honest, accurate, and transparent account of the study being reported, that no important aspects of the study have been omitted, and that any discrepancies from the study as planned have been explained.
